# Chiral Discrimination of Mexiletine Enantiomers by Capillary Electrophoresis Using Cyclodextrins as Chiral Selectors and Experimental Design Method Optimization

**DOI:** 10.3390/molecules27175603

**Published:** 2022-08-31

**Authors:** Melania Cârcu-Dobrin, Gabriel Hancu, Lajos Attila Papp, Ibolya Fülöp

**Affiliations:** 1 Department of Pharmaceutical and Therapeutic Chemistry, Faculty of Pharmacy, University of Medicine, Pharmacy, Science and Technology “George Emil Palade” of Târgu Mureș, 540142 Târgu Mureș, Romania; 2 Department of Toxicology and Biopharmacy, Faculty of Pharmacy, University of Medicine, Pharmacy, Science and Technology “George Emil Palade” of Târgu Mureș, 540142 Târgu Mureș, Romania

**Keywords:** mexiletine, capillary electrophoresis, cyclodextrins, chiral separation, experimental design, molecular modeling

## Abstract

Mexiletine (MXL) is a class IB antiarrhythmic agent, acting as a non-selective voltage-gated sodium channel blocker, used in therapy as a racemic mixture *R*,*S*-MXL hydrochloride. The aim of the current study was the development of a new, fast, and efficient method for the chiral separation of MXL enantiomers using capillary electrophoresis (CE) and cyclodextrins (CDs) as chiral selectors (CSs). After an initial CS screening, using several neutral and charged CDs, at four pH levels, heptakis-2,3,6-tri-O-methyl-β-CD (TM-β-CD), a neutral derivatized CD, was chosen as the optimum CS for the enantioseparation. For method optimization, an initial screening fractional factorial design was applied to identify the most significant parameters, followed by a face-centered central composite design to establish the optimal separation conditions. The best results were obtained by applying the following optimized electrophoretic conditions: 60 mM phosphate buffer, pH 5.0, 50 mM TM-β-CD, temperature 20 °C, applied voltage 30 kV, hydrodynamic injection 50 mbar/s. MXL enantiomers were baseline separated with a resolution of 1.52 during a migration time of under 5 min; *S*-MXL was the first migrating enantiomer. The method’s analytical performance was verified in terms of precision, linearity, accuracy, and robustness (applying a Plackett–Burman design). The developed method was applied for the determination of MXL enantiomers in pharmaceuticals. A computer modeling of the MXL-CD complexes was applied to characterize host–guest chiral recognition.

## 1. Introduction

Mexiletine (MXL) [1-(2,6-dimethylphenoxy)propan-2-amine], is an antiarrhythmic agent belonging to class IB antiarrhythmics that acts at the sodium channel level. It reduces ventricular cardiac automaticity by shortening the potential of action and refractory period [1]. It is mostly prescribed for the treatment of recurrent ventricular tachycardia, especially if it has been preceded by an acute myocardial infarction [2]. MXL also exhibits anesthetic effects, as it is structurally related to lidocaine, but, in contrast to lidocaine, MXL is active after oral administration. It may also have anticonvulsant effects. In the past few years, it has been successfully used in different types of neuropathies, skeletal muscle channelopathies, and amyotrophic lateral sclerosis [3].

**Figure 1:**
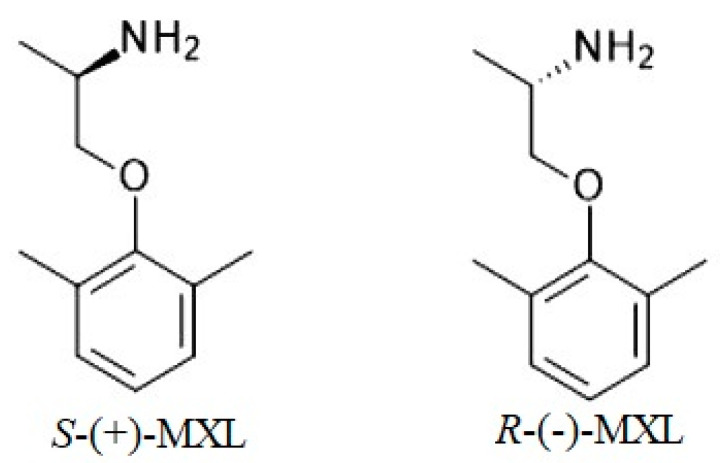
MXL enantiomers’ chemical structures.

Structurally MXL is a primary amine, the 2,6-dimethylphenyl ether of 2-aminopropan-1-ol, and has in its structure a chiral center that generates the existence of two enantiomers, *R*-MXL and *S*-MXL. It is used in therapy as the racemic mixture *R,S*-MXL, which is usually used as a hydrochloride salt [4]. The chemical structures of the two enantiomers are presented in Figure 1.

*R*-(-)-MXL shows higher antiarrhythmic activity than *S*-(+)-MXL, in terms of sodium channel blocking, membrane stabilization, and a reduction in cardiac excitability [5,6]. Moreover, the percentage of *R*-MXL binding to human serum proteins is significantly higher than that of the binding of *S*-MXL [7]. The renal excretion of *R*-MXL is higher than that of *S*-MXL; while the AUC (area under the plasma concentration versus time curve) of *S*-MXL is significantly higher than that of *R*-MXL [8]. MXL undergoes stereoselective aliphatic and aromatic hydroxylation in human microsomes; the aromatic hydroxylation is preferred for *S*-MXL, while the aliphatic hydroxylation predominates for *R*-MXL [9]. MXL undergoes extensive hepatic metabolization; the major metabolites are hydroxymethyl-MXL, *p*-hydroxy-MXL and their corresponding alcohols, hydroxymethyl-MXL-alcohol, and *p*-hydroxy-MXL-alcohol. CYP2D6 mediated aromatic and aliphatic hydroxylation is enantioselective; in vivo studies have suggested that a noncytochrome P450 enzyme also plays a role in the stereoselective disposition of MXL [10]. Based on the pharmacokinetic and pharmacodynamic differences between the MXL enantiomers described above, the development of new methods for their enantioseparation is a subject of pharmaceutical interest.

Capillary electrophoresis (CE) is a powerful and effective technique for the enantioresolution of pharmaceuticals and is being considered today as an alternative and complementary method to the more widely used high-performance liquid chromatographic (HPLC) methods. The advantages of using CE for the chiral separation of pharmaceuticals are related to the high separation efficiency, short analysis time, and relatively low operational costs, taking into consideration the reduced consumption of chemicals (samples, solvents, and chiral selectors (CSs)). Moreover, in CE, usually a direct chiral separation is used simply by adding the CS to the background electrolyte (BGE) [11,12].

Several CSs are available in CE (cyclodextrins (CDs), macrocyclic antibiotics, proteins, crown ethers, etc.); however, the most efficient and frequently used by far are the CD derivatives. Today there are a large number of CD derivatives available as CSs in CE, including native and derivatized as well as neutral and ionized ones. The advantages of using CDs as CSs are related to the high complexation capacity towards many pharmaceuticals, relatively good solubility in aqueous BGE, low UV absorption, and commercial availability [13,14].

CD derivatives have established themselves as CSs in CE because of their ability to interact stereoselectively with many chiral analytes via intermolecular interactions, which generate complexes with different mobilities than the uncomplexed analyte. CD-based enantiorecognition methods are difficult to characterize because guest molecules may interact with CD macrocycles by inclusion in the CD hydrophobic cavity, but CD–guest interactions may also involve the exterior surface of the CD. Several variables can influence CD conformation and recognition ability, including CD structure, as well analyte structure, and separation medium characteristics [15]. Intensive study has been conducted over the last decade to better understand the chiral recognition processes involving CDs. Significant advances were made based on a multidisciplinary approach that included separation and spectroscopic methods, X-ray crystallography, and molecular modeling [15,16].

Traditionally, for analytical method development, the “one-factor-at-a-time” (OFAT) strategy was used, in which one analytical parameter is varied while the others are maintained at a constant; however, this technique has some limitations. The use of design of experiments (DoE) in analytical method development involves multifactorial experiments that lead to optimized solutions by performing the minimum required experiments. DoE is also used to establish factor-response relationships which further helps in identifying the most significant parameters by saving time, effort, and operational costs [17,18].

Based on an extensive literature review, we found only one article describing a chiral separation method by CE of MXL enantiomers using CDs as the CS, published in 1998 by Kang and Ou. In this study, MXL enantiomers were baseline separated using heptakis-2,3,6-tri-O-methyl-β-CD (TM-β-CD) as CSs and a 40 mM tris–phosphoric acid BGE at pH 2.5. Baseline separation was achieved with a resolution of 2.2 and a migration time of over 12 min; however, the method was developed using the OFAT approach, and no validation studies were made [19].

Bonato et al. used MXL together with other basic drugs as model substances (disopyramide, fluoxetine, praziquantel) to verify the enantioselective capacity of sulfated-β-CD (S-β-CD), alone or in dual CS systems. The method was applied for the enantioseparation of MXL and its main metabolites (hydroxymethyl-MXL, *p*-hydroxy-MXL) using a phosphate BGE at pH 7.0 and 2% S-β-CD as the CS. MXL and its metabolites are protonated at pH 7.0, where the analysis was conducted; therefore, the electrostatic interaction with the negatively charged CD may explain the enantioresolution [20].

MXL enantioseparation was performed by CE using two types of serum albumin (human serum albumin-HSA, porcine serum albumin-PSA) in combination with a partial filling technique. Separation was performed at pH 7.4, at which MXL is positively charged and migrates towards the cathode, whereas both HSA and PSA migrated towards the anode, contrary to the electroosmotic flow (EOF). After evaluation of the chiral interactions, HSA showed higher selectivity for MXL over PSA [21]. Xu et al. studied the conformational change of HSA through its binding to MXL by CE. By reversible binding, HSA interacts differentially with MXL enantiomers; the binding interaction is mostly hydrophobic in nature [22]. However, the use of proteins as CSs in CE has some disadvantages related to the adsorption of protein to the capillary wall and the absorption of UV light at the detection wavelength.

Several direct and indirect HPLC methods were published for the chiral separation of MXL enantiomers. For MXL enantioseparation through an indirect approach, several chiral derivatizing reagents have been used: Sanger reagent (2,4-dinitrofluorobenzene) [23], (*S*,*S*)-O,O’-di-p-toluoyl tartaric acid anhydride [24], (*S*)-naproxen [24,25], cyanuric chloride [26], (*S*)-(-)-(N)-trifluoroacetyl-prolyl chloride, (1*S*)-(-)-camphanic chloride [27], and Marfey’s reagent (1-fluoro-2,4-dinitrophenyl-5-*l*-alanine amide) [24,28]. MXL enantioseparation by HPLC was resolved also through a direct approach using different chiral stationary phases (CSPs): Pirkle-type ionic CSP (based on (*R*)-(-)-3,5-dinitrobenzoylphenylglycine) [29], Chiralpak AD CSP based on a carbamoyl derivative of amylose (amylose tris(3,5-dimethylphenylcarbamate) [30,31], and crown ether-based CSP (1,1-binaphthyl crown ether, (18-crown-6)-2,3,11,12-tetracarboxylic acid) [32,33].

Taking the data published in the literature into consideration, our aim was to develop a new, fast, and efficient method for the chiral separation of MXL enantiomers by CE using CDs as chiral selectors and applying DoE strategies for method optimization.

## 2. Results

### 2.1. Preliminary Analysis

MXL is a primary amine with a pKa value of 9.14 and, consequently, is positively charged in an acidic medium [34]. To evaluate the electrophoretic behavior of MXL in an achiral environment, the pH of the BGE was varied over a range of 2.5–9, using four pH levels: 2.5, 5.0, 7.0, and 9.0 and a 50 mM phosphate BGE.

The migration times decreased while increasing the pH of the BGE solution; however, at pH values over 7.0, MXL migrates close to or even together with EOF due to low ionization of the analyte in basic BGE.

The initial CD screening was performed in a 50 mM phosphate BGE at four pH levels (2.5, 5.0, 7.0, and 9.0) by adding 10 mM neutral CDs (α-CD, β-CD, γ-CD, hydroxypropyl-β-CD-HP-β-CD, methylated β-CD-M-β-CD, heptakis(2,6-di-O-methyl)-β-CD-DM-β-CD, heptakis(2,3,6-tri-O-methyl)-β-CD-TM-β-CD) and 5 mM ionized CDs (carboxymethyl-β-CD-CM-β-CD, sulphated β-CD-S-β-CD, sulphobutylether-β-CD-SBE-β-CD), respectively, in the BGE. In the case of charged CDs, lower concentrations were used than in the case of neutral ones to avoid an elevated current in the capillary.

Chiral interactions were observed between MXL and two neutral CD derivatives: heptakis 2,3,6-tri methoxy-β-CD (TM-β-CD), heptakis 2,6-di methoxy-β-CD (DM-β-CD) at pH levels 2.5 and 5.0. The initial chiral resolutions were higher for TM-β-CD, as in the case of DM-β-CD only a small peak splitting was observed. Moreover, the chiral resolutions were higher at pH 5.0 in comparison with pH 2.5 for both CDs. Based on the resolution, peak amplitude, and peak shapes, TM-β-CD was chosen as the optimal CS for further method development at pH 5.0.

The preliminary results confirm the supposition of Kang and Ou [19], as methoxy groups in the CDs structure are thought to be essential for racemic MXL chiral recognition. The substitution of 2- and 3-hydroxy groups of β-CD with methoxy substituents seem to improve the chiral recognition of MXL enantiomers.

TM-β-CD is a neutral derivatized CD without own electrophoretic mobility; consequently, the electrophoretic mobility of the MXL-TM-β-CD complex will be influenced by the ionization of MXL.

### 2.2. Method Optimization

Screening designs are used as the first step in the optimization of an analytical method because they allow the analysis of many factors with a relatively small number of experiments. A fractional factorial design was applied, by fractionating a full factorial 2^k^ design into a 2^k − p^ design, where k is the total number of factors introduced in the design, while p is the size of the fraction [17,18].

In our study, six experimental parameters were taken into consideration: BGE concentration, BGE pH, CD concentration, temperature, voltage, and injection pressure. We performed a fractional design with 2^6 − 3^ = 2^3^ = 8 experiments with four center point replicates that generated a matrix with 12 experiments. Replicated center point experiments can be used to estimate the experimental error and to assess the curvature of the model. All parameters were varied from the mean value on a specific range with a lower and a superior value, and the monitored responses were the chiral resolution (R) and migration times of the second migrating enantiomer (t). An overview of the experiments and the results obtained in the factorial screening design is presented in Table 1.

In the initial stage of DoE, screening designs are frequently used to identify the significant input factors and exclude the less significant ones. Therefore, an initial regression model is constructed, and then the significance of the model coefficients is determined. This can be performed by using graphical and/or statistical methods.

As a graphical tool, Pareto charts were used to identify the most significant parameters on the chiral discrimination of the MXL enantiomers; CD concentration, BGE concentration, and injection pressure had a significant influence on chiral resolution, while voltage, BGE concentration, and CD concentration had a pronounced effect on migration times.

The analysis of variance (ANOVA), performed as a statistical evaluation of the regression model, delivered the same results as those obtained by the graphical approach. The insignificant factors were removed one by one to improve the model, and the following regression equations were obtained:

Resolution (R) = 0.94 − 0.13 × B + 0.15 × C − 0.12 × F(1)

Migration time t (min) = 4.18 + 0.38 × B − 0.31 × C − 0.92 × E(2)

where B represents the BGE concentration, C represents the CD concentration, E represents the voltage, and F represents the injection pressure.

In accordance with the results obtained in the fractional factorial screening step, BGE concentration, CD concentration, and voltage were chosen as factors that significantly interfere with MXL enantioseparation and were further taken into consideration in the optimization design. The three other factors were kept constant: BGE pH—5.0, temperature—20 °C, injection pressure—50 mbar/s.

A face-centered central composite design (FCCD) with three variables was used for method optimization based on the results of the screening design. The same experimental responses were monitored as in the screening design: chiral resolution (R) and migration time of the second migrating enantiomer (t). The FCCD matrix consisted of 15 experiments with five center points. Experiments were performed in random order to avoid systematic errors. An overview of the experiments and results obtained in the FCCD is presented in Table 2.

An initial quadratic regression model, including linear, interaction, and second order terms, was applied, and the ANOVA was performed to identify the statistically significant model terms. The factors considered insignificant were removed one by one. In the case of chiral resolution, as initially predicted through the factorial screening design, voltage did not represent a significant factor and was completely removed from the equation.

The following recalculated regression equations were obtained, after insignificant parameter reduction:

Resolution (R) = 1.26 + 0.057 × A + 0.13 × B − 0.057 × B^2^ (3)

Migration time t (min) = 4.39 + 0.29 × A + 0.19 × B − 0.38 × C + 0.12 × AB + 0.16 × AC + 0.25 × BC + 0.11 × A^2^ + 0.043 × B^2^ + 0.048 × C^2^ (4)

where A represents the BGE concentration, B represents the CD concentration, and C represents the voltage.

During regression model adjustment, the values of three types of determination coefficients (*R^2^*, *R^2^_adj_*, and *R^2^_pred_*) were followed as performance indicators. According to the values *R^2^* 0.9381 and *R^2^_adj_* 0.9132 for R and *R^2^* 0.9986 and *R^2^_adj_* 0.9960 for t, respectively, both models fit the experimental results. Moreover, *R^2^_pred_* was in reasonable agreement with *R^2^_adj_* (0.8921 vs. 0.9132 for R and 0.9909 vs. 0.9960 for t, respectively).

**Figure 2:**
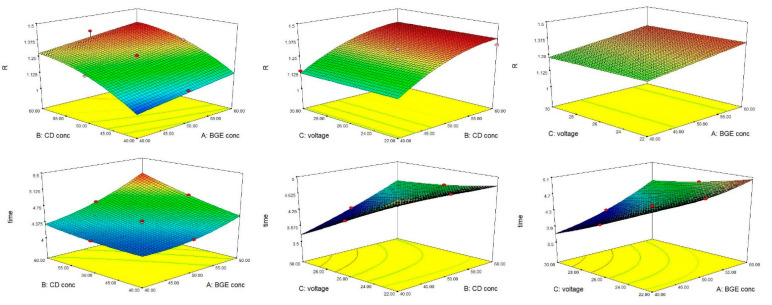
3D response surface plots for MXL chiral separation (analytical responses: R—chiral resolution, time—migration time).

Three-dimensional response surface plots were created for each analytical response; the graphs illustrate the interactions between two factors and their influence on the monitored response, while the third factor is kept constant. The 3D graphs for both analytical responses are presented in Figure 2.

Desirability functions are usually designed to achieve different criteria; to maximize, minimize, and optimize two or more analytical responses [17,18]. Following optimization using the FCCD experimental design, the target parameters (BGE concentration, CD concentration, voltage) were defined using the numerical optimization function of the software with the aim of maximizing resolution and minimizing analysis time (Table 3).

**Figure 3:**
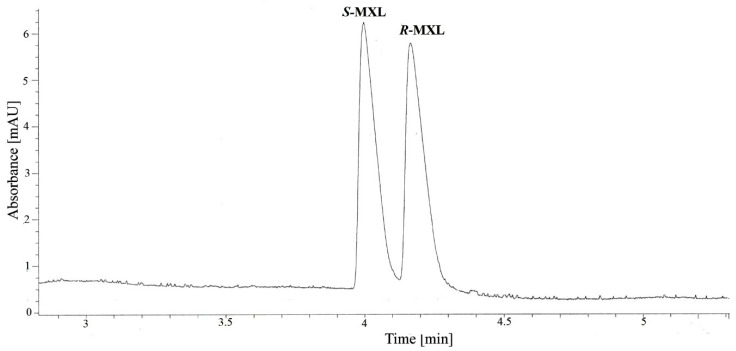
Chiral separation of MXL enantiomers under optimized analytical conditions (experimental conditions: 60 mM phosphate BGE, pH 5.0, 50 mM TM-β-CD, 20 °C, 30 kV, 50 mbar/s, 210 nm).

The chiral separation of MXL enantiomers was achieved by applying the following optimized conditions: 60 mM phosphate BGE, BGE pH 5.0, 50 mM TM-β-CD, temperature 20 °C, applied voltage 30 kV, hydrodynamic injection 50 mbar/s. The baseline separation of MXL enantiomers was obtained with a resolution of 1.52 in less than 5 min. The migration order was determined by spiking the racemate with a pure enantiomer solution and using the differential migration time of the enantiomers; the migration order was *S*-MXL followed by *R*-MXL. A typical electropherogram for MXL chiral separation under optimized conditions is presented in Figure 3.

### 2.3. Molecular Docking of MXL-CD Complexes

Molecular docking was applied to verify the migration order of the enantiomers obtained experimentally and to obtain information on the interaction energy as well as the preliminary data of the geometry of the inclusion complexes.

**Figure 4:**
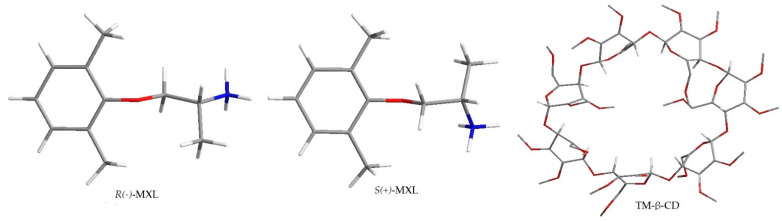
Energy-minimized models of MXL enantiomers and TM-β-CD (white—H, red—O, grey—C, blue—N).

The energy-minimized models for MXL enantiomers and TM-β-CD are presented in Figure 4.

Calculation with 1:1 MXL-CD stoichiometry provided an appropriate free binding energy (ΔG) to conclude the formation of inclusion complexes between the host and guest molecules in all 2 × 100 configurations analyzed, with an average ΔG value of −3.92 ± 0.201 kcal/mol for the *R*-MXL-CD complex and ΔG= −3.85 ± 0.220 kcal/mol for the *S*-MXL-CD complex, where a higher negative value indicates the formation of a more stable complex. The difference between the binding energies of the all-possible host–guest configurations for both enantiomers (*n* = 100) is statistically significant (0.047, *t*-test, one-tailed for two independent means) in favor of *R*-MXL-CD complexes. The average binding constant (K_b_) is higher in the case of the complex formed with *R*-MXL than with *S*-MXL (1.58 ± 0.57 mM and 1.48 ± 0.54 mM).

Regarding the conformation of the most stable complexes, the propan-2-ammonium moiety of MXL is oriented to the narrower end of the TM-β-CD cone. A hydrogen bond is formed between the oxygen in the glucopyranose unit of the TM-β-CD as a hydrogen bond acceptor and one of the hydrogens in the ammonium moiety of MXL with a length of 2.109 Å and an angle of 143.89° in the case of the *R*-MXL-CD complex.

**Figure 5:**
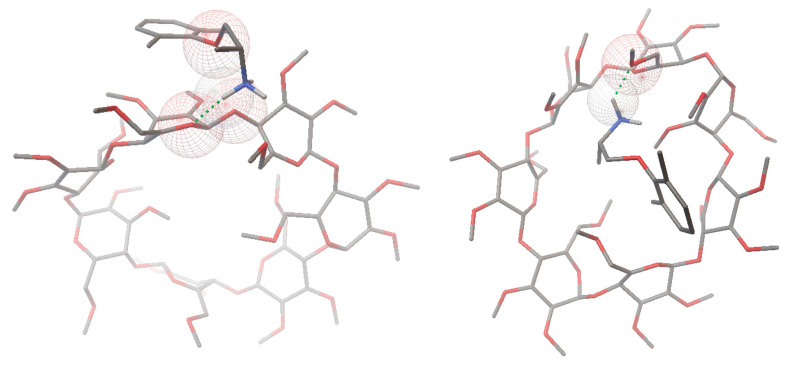
Three-dimensional models of the *R*-MXL-CD (**left**) and *S*-MXL-CD (**right**) complexes with the highest binding energy (Green dots show the hydrogen bonds. Spheres mark the participating atoms. Grey is carbon, red is oxygen, and dark blue is nitrogen).

In the case of the S-MXL-CD complex, a hydrogen bond is identified by the used software, linking the oxygen at the 6-O-methyl part of the TM-β-CD and the ammonium moiety of MXL with a length of 1.842 Å and an angle of 130.05° (Figure 5).

Other types of energies, such as van der Waals force, electrostatic, and desolvation potentials, hold the guest and host molecules together. Based on the data obtained from the docking study, the first migrating enantiomer could be identified as *S*-MXL, and the second migration enantiomer as *R*-MXL, respectively. Taking the complexity of the chiral separation mechanism in CE into consideration, in addition to the stability of the enantiomer–CD complexes, other parameters could also influence the migration order as differences in the electrophoretic mobility of the complexes. The migration order established by molecular docking was in accordance with the experimental results.

### 2.4. Analytical Performance

The analytical performance of the CE method developed for MXL enantioseparation has been verified in terms of precision and reproducibility (intra-day and inter-day), linearity, accuracy, and robustness.

Intra-day precision was determined by injecting a sample of 0.15 mg/mL racemic mixture of MXL six times on the same day, while inter-day precision was determined by injecting a sample of the same concentration six times on three consecutive days. Relative standard deviations (RSDs) were calculated for the migration times, peak area, and peak height for both enantiomers.

Calibration curves were obtained by measuring standard solutions (*n* = 3) at nine different concentrations over a specific concentration range (0.015–0.3 mg/mL). Correlation factors of 0.9998 for both enantiomers demonstrate the good linearity of the method.

The limit of detection (LOD) and limit of quantification (LOQ) were estimated as follows: the standard deviation of the regression equation reported to the regression equation’s slope multiplied by 3.3 for the LOD and by 10 for the LOQ.

Recovery experiments utilizing the common standard addition technique were carried out to evaluate the method’s accuracy. An appropriate amount of MXL capsule powder was weighed and spiked with a quantity of MXL standard; each sample was examined in triplicate. The good recovery values demonstrate good method accuracy.

The data obtained for precision, linearity, and accuracy testing is presented in Table 4.

To verify the method robustness, a Plackett–Burman design was applied. Plackett–Burman designs are unique forms of two-level fractional factorial designs that permit the investigation of up to N-1 input variables using N experiments [17,18]. The following four parameters were taken into consideration: BGE concentration (55, 60, 65 mM), CD concentration (48, 50, 52 mM), BGE pH (4.5, 5, 5.5), and temperature (19, 20, 21 °C), while the analytical responses were the chiral resolution (R) and the migration time of the second enantiomer (t) (Table 5). Based on the ANOVA statistical evaluation performed, it was concluded that no significant influence of any of the studied parameters was observed, and the method robustness was good.

The optimized method was applied for the determination of MXL enantiomers from a pharmaceutical product; capsules with a concentration of 100 mg MXL were used for the determination. The concentrations of the two enantiomers were determined using the optimized CE method and agreed with the composition of a racemic mixture with a 1:1 enantiomer ratio (Table 6).

## 3. Conclusions

A rapid, simple, and cost-effective method was developed for the chiral separation MXL enantiomers by CE using CDs as the CS. Based on a complex CD screening, TM-β-CD, a derivatized neutral CD, was chosen as the optimal CS, and was added in a phosphate BGE at pH 5.0 for the enantioseparation.

An initial screening fractional factorial design was applied to identify the significant parameters that interfere with the enantioseparation. The significance ranking of analytical parameters was performed using Pareto charts as well as the ANOVA; BGE concentration, CD concentration, and voltage were chosen as significant factors to be introduced in the optimization design. For the method optimization, an FCCD was applied, and three levels were varied for each studied parameter. Baseline chiral separation of the MXL enantiomers was achieved with a chiral resolution of 1.52 and a migration time of under 5 min.

The analytical performance of the method was evaluated in terms of precision, reproducibility, linearity, accuracy, and robustness by applying a Plackett–Burman DoE. The applicability of the method was verified by determining the ratio of the two enantiomers from a pharmaceutical product (capsules).

The chiral discrimination mechanism was elucidated by applying molecular modeling calculations; the migration order of the enantiomers was in accordance with the one obtained experimentally.

Compared with the previously published method of Kang and Ou [19], our method offers a shorter migration time (under 5 min vs. above 12 min), but with a lower chiral resolution (1.52 vs. 2.20). Our approach is less sensitive than HPLC enantioseparation methods of MXL, but it still provides quick analysis times, good separation efficiencies, and minimum analyte, CS, and solvent usage, being an approach closer to “green chemistry” principles.

## 4. Materials and Methods

### 4.1. Chemicals and Reagents

A pharmaceutical-grade racemic mixture of MXL hydrochloride (Piramal Enterprises Ltd., Mumbai, India) and pure enantiomer *R*-MXL hydrochloride (Cayman Chemical, Ann Arbor, MI, USA) was used in the analysis. The following analytical grade reagents were used: phosphoric acid 85%, disodium hydrogen phosphate (Merck, Darmstadt, Germany), monosodium hydrogen phosphate (Alfa Aesar, Karlsruhe, Germany), sodium hydroxide (Alfa Aesar, Karlsruhe, Germany), and methanol (Merck, Darmstadt, Germany). Deionized water was prepared by using a Milli-Q apparatus (Millipore, Burlington, MA, USA).

The following CDs were used as the CS in the screening process: neutral natural CDs—α-CD, β-CD, γ-CD (Cyclolab, Budapest, Hungary); neutral derivatized CDs—hydroxypropyl-β-CD (HP-β-CD) (Sigma-Aldrich, Darmstadt, Germany), methylated β-CD (M-β-CD) (Cyclolab, Budapest, Hungary), heptakis(2,6-di-O-methyl)-β-CD (DM-β-CD), and heptakis(2,3,6-tri-O-methyl)-β-CD (TM-β-CD) (Sigma-Aldrich, Darmstadt, Germany); ionizable derivatized anionic CDs—carboxymethyl-β-CD (CM-β-CD), sulphated β-CD (S-β-CD) (Sigma-Aldrich, Darmstadt, Germany), and sulphobutylter-β-CD (SBE-β-CD) (Cydex Pharmaceuticals, Lenexa, KX, USA).

To determine the concentration of enantiomers in pharmaceutical products, Mexitil 100 mg capsules (Boehringer, Ingelheim, Germany) were used.

### 4.2. Instrumentation

The determinations were performed on an Agilent 1600 CE system (Agilent Technologies, Waldbronn, Germany) equipped with DAD (diode array) detector. For the separations, a silica capillary of 48 cm length (effective length 40 cm) and an internal diameter of 50 µm (Agilent Technologies, Waldbronn, Germany) was used. Chemstation 7.01 software (Agilent Technologies, Waldbronn, Germany) was used for electropherogram processing. The pH of the solutions was determined on a Terminal 740 pH meter (Inolab, Germany).

The method’s analytical performance was verified using Microsoft Excel 365 software (Microsoft, Redmond, WA, USA), while the DoE optimization was carried out using Design Expert 7.0 software (State-Ease, Minneapolis, MN, USA).

To estimate the migration order for the two enantiomers, a molecular docking study was performed, considering that the difference in binding energy between the guest molecules (*R*-MXL and *S*-MXL) and the host molecule (TM-β-CD) results in a different migration time. The 2D coordinates of MXL and TM-β-CD were imported from PubChem database, and the 3D structure was built and geometrically optimized at the protonation state, which correspond to pH = 5, used at the chiral separation under the optimized conditions (MM+, Polak–Ribiere algorithm, RMS < 0.01), using the HyperChem 8.0 software (Hypercube, Gainesville, FL, USA) and saved in mol format. Using the OpenBabelGUI 3.1.1 interface, the mol files were converted into the pdb format. Molecular docking simulations with AutoDock 4.2.6 (The Scripps Research Institute, La Jolla, CA, USA) were performed using default parameters, considering flexible MXL and rigid TM-β-CD. Grid boxes, with dimensions of 50 × 50 × 50 Å and a grid spacing of 0.375 Å, were placed where MXL occupied the center of the box. The number of the Lamarckian genetic algorithm was set to 1000 runs [35].

### 4.3. Electrophoretic Conditions

The capillary conditioning was carried out with 0.1 M NaOH solution for 30 min, followed by purified water for 15 min, and then BGE for another 15 min. The capillary was preconditioned between measurements with 0.1 M NaOH solution for 2 min, purified water for 1 min, and BGE for 2 min.

Buffer solutions were prepared by dissolving the appropriate quantity of the constituents in purified water, and pH was adjusted with 1 M phosphoric acid solution or 1M sodium hydroxide solution, when necessary. Stock solutions of the racemic mixture of MXL hydrochloride were prepared in methanol and diluted with the same solvent to the appropriate concentrations.

All the solutions were filtered through a 0.45 µm pore diameter membrane filter and placed in an ultrasonic bath for degassing for a minimum of 5 min before use.

The injection was hydrodynamically performed at the anodic end of the capillary, while detection occurred at the cathodic end at 210 nm wavelength.

To evaluate the analyte–CS interactions, the separation factor and resolution were taken into consideration. The separation factor (α), was calculated from the ratio of the migration times of the two enantiomers, while the resolution was calculated based on the formula: R = 2(t_2_ − t_1_)/(w_1_ + w_2_), where t_1_ and t_2_ represent the migration times, and w_1_ and w_2_ represent the peak widths of the two optical isomers.

### 4.4. Pharmaceutical Sample Preparation

For pharmaceutical sample preparation, the contents of ten capsules were weighed and homogenized in a mortar. An amount of powder corresponding to the average mass of one capsule (100 mg MLX hydrochloride) was dissolved in methanol, homogenized in the ultrasonic bath for 5 min, and diluted with methanol to the appropriate concentrations. Electrophoretic conditions were the same as those used in the chiral analysis of the standard.

## Figures and Tables

**Table 1:** Experimental plan and results obtained in the 2^6 − 3^ fractional factorial screening design.

Nr	pH	BGE Conc. [mM]	CD Conc. [mM]	Temperature (°C)	Voltage (kV)	Injection Pressure (mbar/s)	R	t (min)
1	5.00	50.00	40.00	20.00	25.00	40.00	0.95	3.97
2	5.00	50.00	40.00	20.00	25.00	40.00	0.96	3.99
3	6.00	25.00	30.00	15.00	20.00	50.00	0.67	4.38
4	5.00	50.00	40.00	20.00	25.00	40.00	0.93	3.96
5	6.00	75.00	50.00	25.00	30.00	50.00	1.18	3.71
6	5.00	50.00	40.00	20.00	25.00	40.00	0.98	4.01
7	6.00	75.00	30.00	25.00	20.00	30.00	1.13	5.15
8	4.00	75.00	50.00	15.00	20.00	50.00	1.17	6.23
9	4.00	25.00	50.00	25.00	20.00	30.00	1.15	4.82
10	4.00	25.00	30.00	25.00	30.00	50.00	0.55	2.89
11	4.00	75.00	30.00	15.00	30.00	30.00	1.09	3.31
12	6.00	25.00	50.00	15.00	30.00	30.00	1.15	3.37

**Table 2:** Experimental plan and results obtained in the FCCD optimization design.

Nr	BGE Conc. [mM]	CD Conc. [mM]	Voltage [kV]	R	t [min]
1	50.00	50.00	26.00	1.30	4.40
2	60.00	50.00	26.00	1.36	4.85
**3**	50.00	50.00	26.00	1.29	4.42
4	50.00	60.00	26.00	1.42	4.67
5	40.00	50.00	26.00	1.22	4.27
6	60.00	60.00	22.00	1.39	5.20
7	60.00	40.00	30.00	1.19	4.16
8	50.00	50.00	30.00	1.36	4.09
9	50.00	50.00	22.00	1.32	4.87
10	50.00	40.00	26.00	1.18	4.30
11	40.00	60.00	30.00	1.33	4.13
12	50.00	50.00	26.00	1.29	4.45
13	40.00	40.00	22.00	1.02	5.08
14	50.00	50.00	26.00	1.29	4.45
15	50.00	50.00	26.00	1.31	4.46

**Table 3:** Software solution vs. experimental results for the chiral separation of MXL.

Results	BGE Concentration [mM]	CD Concentration [mM]	Voltage [kV]	R	t [min]
Software Solution	58.48	49.79	28.95	1.48	4.54
Experimental Results	60.00	50.00	30.00	1.52	4.26

**Table 4:** Analytical performance of the optimized method.

MXL		*S*-MXL	*R*-MXL
**Precision**			
Intra-day precision (sample concentration = 0.15 mg/mL, *n* = 6)	RSD%, migration time	0.01	0.01
	RSD%, peak area	0.18	0.19
	RSD%, peak height	0.16	0.15
Inter-day precision (sample concentration = 0.15 mg/mL, *n* = 18)	RSD%, migration time	0.05	0.05
	RSD%, peak area	0.62	0.88
	RSD%, peak height	0.29	0.34
**Accuracy (recovery %)**			
0.15 mg/mL		101.07	99.82
0.075 mg/mL		101.52	99.18
0.025 mg/mL		102.64	98.26
**Linearity**			
Regression equation (0.015–0.3 mg/mL)		y = 338.77x + 0.476	y = 336.19x + 0.2673
Coefficient of correlation		0.9998	0.9998
LOD (mg/mL)		0.0052	0.0047
LOQ (mg/mL)		0.0159	0.0142

**Table 5:** Plackett–Burman design for robustness testing.

Nr	BGE Conc. [mM]	CD Conc. [mM]	pH	Temperature [°C]	R	t [min]
1	60.00	50.00	5.00	20.00	1.52	4.26
2	65.00	48.00	4.50	19.00	1.47	4.35
3	65.00	48.00	5.50	21.00	1.46	4.25
4	60.00	50.00	5.00	20.00	1.5	4.3
5	60.00	50.00	5.00	20.00	1.51	4.28
6	65.00	52.00	4.50	19.00	1.48	4.37
7	65.00	52.00	4.50	21.00	1.48	4.32
8	55.00	48.00	5.50	19.00	1.46	4.27
9	60.00	50.00	5.00	20.00	1.52	4.3
10	55.00	48.00	4.50	19.00	1.46	4.26
11	55.00	52.00	5.50	21.00	1.49	4.33
12	55.00	48.00	4.50	21.00	1.47	4.25
13	65.00	52.00	5.50	19.00	1.49	4.28
14	55.00	52.00	5.50	19.00	1.48	4.27
15	65.00	48.00	5.50	21.00	1.49	4.32
16	55.00	52.00	4.50	21.00	1.47	4.34

**Table 6:** MXL enantioselective determination from pharmaceutical formulation.

Pharmaceutical Product	Declared Enantiomers Quantity (mg)		Found Enantiomer Quantity (mg) ± SD (*n* = 3)	
	*S*-MXL	*R*-MXL	*S*-MXL	*R*-MXL
Mexitil capsules (100 mg MXL)	50	50	49.82 ± 0.34	49.75 ± 0.29

## Data Availability

Not applicable.
